# Magnetic Resonance Imaging Features of Plantar Vein Thrombosis

**DOI:** 10.3390/diagnostics14121215

**Published:** 2024-06-08

**Authors:** Frederico Celestino Miranda, Adham do Amaral e Castro, Ariadne Moura Obrigon, Alexandre Leme Godoy-Santos, Durval do Carmo Barros Santos, Laercio Alberto Rosemberg, Atul Kumar Taneja

**Affiliations:** 1 Hospital Israelita Albert Einstein, São Paulo 05652-900, Brazil; fcmmsk@gmail.com (F.C.M.); adham.castro@gmail.com (A.d.A.e.C.); ariadne.m10@gmail.com (A.M.O.); alexandrelemegodoy@gmail.com (A.L.G.-S.); durvalcs@einstein.br (D.d.C.B.S.); laercio.rosemberg@einstein.br (L.A.R.); 2 Department of Imaging Diagnosis, Universidade Federal de São Paulo, São Paulo 04024-002, Brazil; 3 Faculdade de Medicina, Universidade de São Paulo, São Paulo 05403-010, Brazil; 4 Department of Radiology, UT Southwestern Medical Center, Dallas, TX 75390, USA

**Keywords:** DVT, MRI, Doppler, deep venous thrombosis, foot pain, metatarsalgia, thrombophlebitis

## Abstract

Plantar vein thrombosis (PVT) is an underdiagnosed condition affecting the deep plantar veins, with challenging clinical diagnosis, often presenting with non-specific symptoms that mimic other foot pathologies. This study assessed the magnetic resonance imaging (MRI) features of patients diagnosed with PVT to contribute to the understanding of this condition. We performed the comprehensive analysis of a substantial dataset, including 112 patients, with a total of 130 positive MRI scans (86 of the forefoot and 44 of the ankle) presenting with PVT. Upon evaluating all the veins of the feet, we observed a higher frequency of involvement of the lateral plantar veins (53.1%) when compared to the medial veins (3.8%). The most affected vascular segments in the forefeet were the plantar metatarsal veins (45.4%), the plantar venous arch (38.5%), and the plantar communicating veins (25.4%). The characteristic findings on MRI were perivascular edema (100%), muscular edema (86.2%), venous ectasia (100%), perivascular enhancement (100%), and intravenous filling defects (97.7%). Our study provides valuable insights into the imaging evaluation of PVT and shows that MRI is a reliable resource for such diagnosis.

## 1. Introduction

Plantar vein thrombosis (PVT) is an uncommon venous disorder and an underdiagnosed condition affecting the deep plantar veins, with few studies addressing this entity in the medical literature [1,2,3]. Clinical diagnosis is usually challenging due to the unfamiliarity of this entity with clinicians and surgeons, with many differential diagnoses for plantar foot pain, including plantar fasciitis, Morton’s neuroma, sesamoiditis, plantar fibromatosis, tendon pathologies, and stress fractures, among others [1,2,4]. PVT pathogenesis is uncertain and can be idiopathic in up to 50% of cases [5], or related to local or systemic factors [1,6,7,8]. The “Virchow triad” (venous ectasia, endothelial damage, and inflammation) is recognized as the traditional explanation for the pathophysiological mechanism behind the development of deep venous thrombosis (DVT) [8,9]; however, the possible predisposing factors include coagulation disorders, oral contraceptives, infection, malignancy, airplane travel, trauma, mechanical stress, athletic activity, recent surgery, pos-operative immobilization, and pressure from orthotics [1,2,6,7]. The most common presentation is a non-specific plantar sensation, or moderate-to-severe localized foot pain and swelling [1,6,10].

The true prevalence and incidence of PVT is unknown [10] due to the lack of epidemiological studies, but some studies suggest that plantar vein thrombosis accounts for 10% of patients with DVT overall, compared to 44% with DVT affecting the calf veins [1,5,11].

The standard imaging protocol to diagnose venous thrombosis is ultrasonography (US), but the usual assessment for DVT usually does not include the evaluation of plantar veins [7]. On the other hand, magnetic resonance imaging (MRI) is a reliable tool in clinical practice [1,4,12], with well-established imaging findings for DVT, including intravenous filling defects, venous ectasia, and perivascular soft-tissue edema and enhancement, but dedicated studies for PVT features on imaging are just scarce case reports and case series [1,4].

The treatment for PVT lacks standardization [13]. The options include NSAIDs alone or anticoagulation with compression stockings. Omitting anticoagulants may increase the PVT progression risk, but their use poses a bleeding risk. Clinical thrombophilia assessment is recommended for all PVT patients, similar to DVT evaluation [1].

The purpose of the present study was to evaluate the MRI findings of patients diagnosed with PVT and provide a comprehensive panel of its imaging features.

## 2. Materials and Methods

### 2.1. Ethical Approval

This research was conducted with the approval of the Institutional Review Board of Hospital Israelita Albert Einstein (CAAE: 52636121.8.0000.0071), and informed consent was not required, given its retrospective nature.

### 2.2. Patients

A retrospective search was performed in a database of reports generated for forefoot and ankle MRI findings obtained at our institution from April 2013 to December 2021. To this end, an organizational business intelligence tool was used. Among the forefoot and ankle MR studies performed during this interval, cases were selected by searching for a combination of terms in the impression field of radiological reports, including “thrombosis, thrombophlebitis, thrombus”. The exclusion criteria were suboptimal MRI examination (motion artifacts or incomplete study protocol), evidence of prior foot surgery, infectious process, tumors, the absence of PVT on MRI review despite a positive report, and thrombosis located in other regions of the foot and not located in the deep plantar veins.

The STROBE (Strengthening the Reporting of Observational Studies in Epidemiology) checklist for cross-sectional studies was followed to collect and describe the data [14]. The data were anonymized in the Research Electronic Data Capture (REDCap^®^ plaform, Version: 13.7.5, ©2024, Vanderbilt University, Nashville—TN) [15,16]. The anonymized data from the REDCap^®^ platform were exported to a Microsoft Excel^®^ spreadsheet.

### 2.3. Clinical Data

All the cases had their medical records, radiology reports, and surgical notes reviewed when available. Each subject had their anthropometric, clinical, and imaging findings annotated and tabulated. The clinical data included age (years), gender (male, female), weight (kg), height (m), body mass index (BMI), and time between onset of pain and MRI examination (days).

### 2.4. Imaging Studies

The MRI studies of the forefoot and/or ankle were performed according to the departmental protocol in a prone and supine positions, respectively, and with leg extension with the use of a phased array dedicated coil on 1.5 T or 3.0 T scanners.

The majority of forefoot examinations followed these sequences: sagittal T1-weighted scan (TR/TE, 541.0/8.8; number of excitations [NEX], 2; matrix, 384 × 288; thickness, 3 mm; field of view [FOV], 15 cm), sagittal T2-weighted scan with fat suppression (3879/42; 3; 384 × 288; 3 mm; 15 cm), coronal T2-weighted scan with fat suppression (3620/42; 3; 384 × 307; 3 mm; 15 cm), axial T2-weighted scan with fat suppression (4179/51; 3; 320 × 320; 3 mm; 15 cm), axial T1-weighted scan (658.0/8.77; 2; 384 × 288; 3 mm; 12 cm), and oblique proton-density (PD)-weighted scan (2955/42; 3; 384 × 307; 3 mm; 15 cm).

The majority of ankle examinations followed these sequences: sagittal T1-weighted scan (TR/TE, 607/8; NEX, 2; matrix, 384 × 288; thickness, 3 mm; FOV, 15 cm), sagittal T2-weighted scan with fat suppression (3879/42; NEX, 3; matrix, 384 × 288; thickness, 3 mm; FOV, 15 cm), coronal T2-weighted scan with fat suppression (3620/42; NEX, 3; matrix, 384 × 307; thickness, 3 mm; FOV, 15 cm), axial T2-weighted scan with fat suppression (4179/51; NEX, 3; matrix, 320 × 320; thickness, 3 mm; FOV, 15 cm), axial T1-weighted scan (549.0/9.08; NEX, 1; matrix, 320/256; thickness, 3.5 mm; FOV, 15 cm), and oblique PD-weighted scan (2955/42; NEX, 3; matrix, 384 × 307; thickness, 3 mm; FOV, 15 cm).

### 2.5. Imaging Analysis

Two fellowship-trained musculoskeletal radiologists (15 years’ and 7 years’ experience each, respectively, readers 1 and 2) evaluated the MRI examinations using all the imaging planes on a picture archiving and communications system (PACS).

First, the two radiologists analyzed the images in consensus to determine which cases were positive or negative for PVT. In a second step, readers 1 and 2 independently evaluated the MR images for the subsequent evaluation of interobserver agreement regarding multiple parameters. The results of the most experienced radiologist (reader 1) were used for statistical analysis.

**Figure 1:**
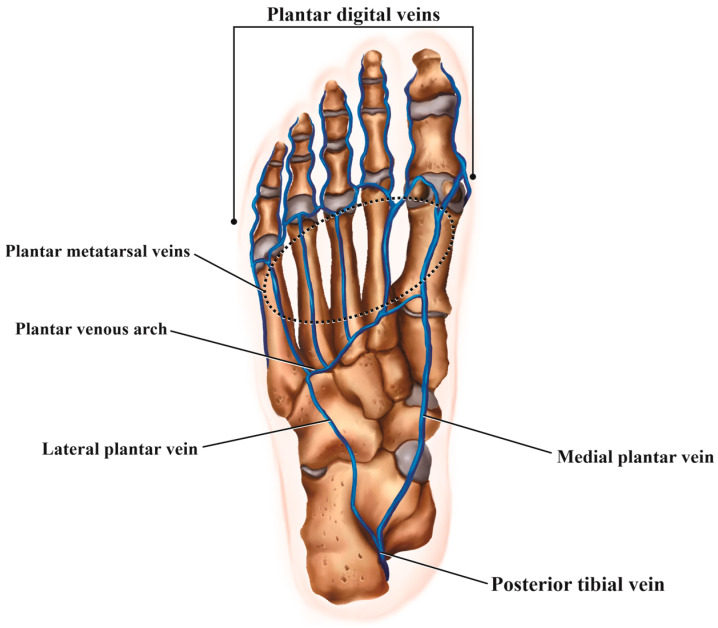
Diagram showing the venous anatomy of the foot seen from the plantar aspect.

**Figure 2:**
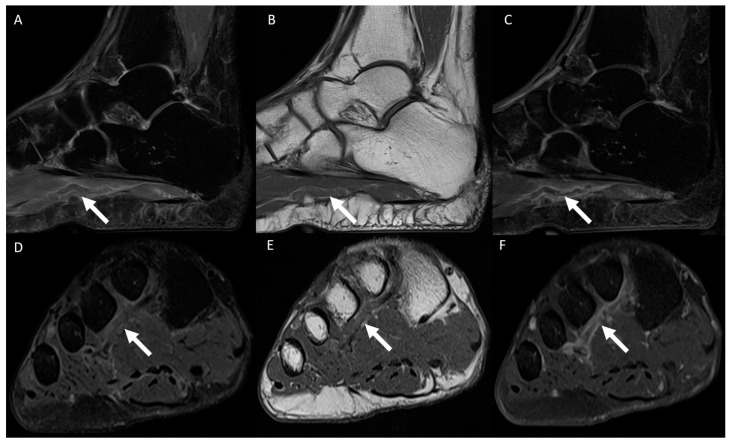
A 71-year-old woman presented with plantar pain of her right foot while walking for the past 7 days. Sagittal MRI images of the ankle, including a T2-weighted fat-suppressed scan (**A**), a T1-weighted scan (**B**), and a post-gadolinium T1-weighted fat-suppressed scan (**C**), revealed thrombosis in the lateral plantar vein, identified by venous dilation, with intraluminal thrombus (arrow in (**A**,**B**)) and an intravenous filling defect (arrow in (**C**)). Additionally, the short-axis plane MRI images of the forefoot, including a T2-weighted fat-suppressed scan (**D**), a T1-weighted scan (**E**), and a post-gadolinium T1-weighted fat-suppressed scan (**F**), exhibited thrombosis in the plantar venous arch, characterized by venous enlargement with intraluminal thrombus (arrow in (**D**,**E**)) and an intravenous filling defect (arrow in (**F**)).

**Figure 3:**
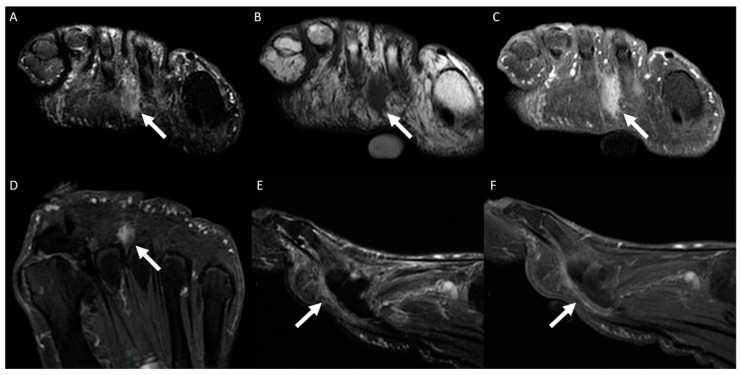
A 77-year-old man presented with left forefoot plantar pain persisting for 30 days. MRI images of the forefoot in short-axis plane, including a T2-weighted fat-suppressed scan (**A**) and a T1-weighted scan (**B**), as well as post-gadolinium T1-weighted fat-suppressed scan (**C**), along with a coronal T2-weighted fat-suppressed scan (**D**) and a sagittal T2-weighted fat-suppressed scan (**E**) and a post-gadolinium T1-weighted fat-suppressed scan (**F**), revealed thrombosis in the plantar metatarsal vein. This condition was characterized by venous dilation, with intraluminal thrombus and an intravenous filling defect (indicated by arrows), mimicking the diagnosis of Morton’s neuroma. Notably, the sagittal plane images (**E**,**F**) clearly depict the extent of thrombus within the metatarsal vein.

**Figure 4:**
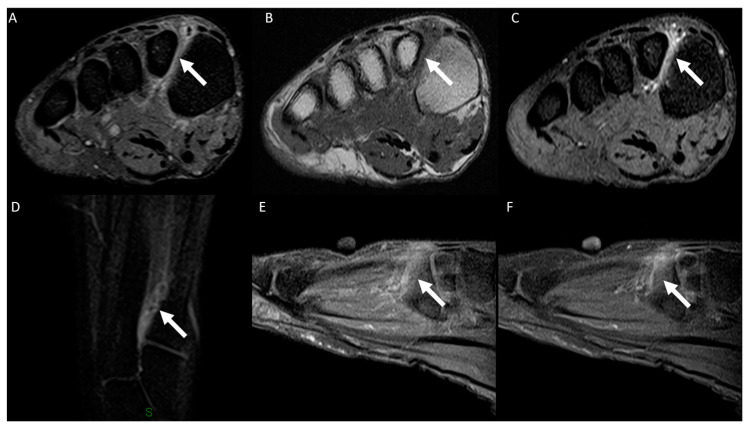
A 54-year-old woman presented with pain in her right foot while walking, persisting for 15 days. Forefoot MRI images in the short-axis plane, including a T2-weighted fat-suppressed scan (**A**) and a T1-weighted scan (**B**), as well as a post-gadolinium T1-weighted fat-suppressed scan (**C**), and in the long-axis plane, a post-gadolinium T1-weighted fat-suppressed scan (**D**), along with a sagittal T2-weighted fat-suppressed scan (**E**) and a post-gadolinium T1-weighted fat-suppressed scan (**F**), revealed thrombosis in the perforating vein of the first space (indicated by arrows). This condition was characterized by venous dilation, with intraluminal thrombus and an intravenous filling defect.

**Figure 5:**
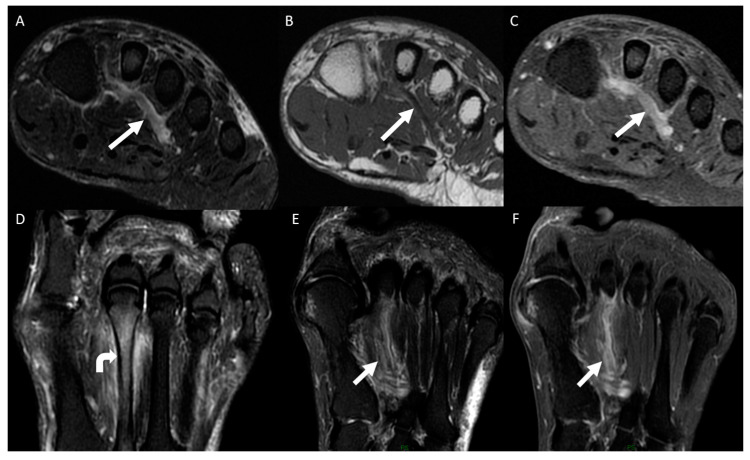
A 64-year-old woman presented with left foot plantar pain experienced while walking, persisting for 5 days. Forefoot MRI images in the short-axis plane, including a T2-weighted fat-suppressed scan (**A**) and a T1-weighted scan (**B**), as well as a post-gadolinium T1-weighted fat-suppressed scan (**C**), and in the long-axis plane, a T2-weighted fat-suppressed scan (**D**,**E**), along with a post-gadolinium T1-weighted fat-suppressed scan (**F**), revealed thrombosis in the metatarsal vein (arrows). This condition was characterized by venous dilation, with intraluminal thrombus and an intravenous filling defect. Image (**D**) displays a stress fracture of the second metatarsal (curved arrow), along with bone marrow edema and adjacent soft tissue edema.

**Figure 6:**
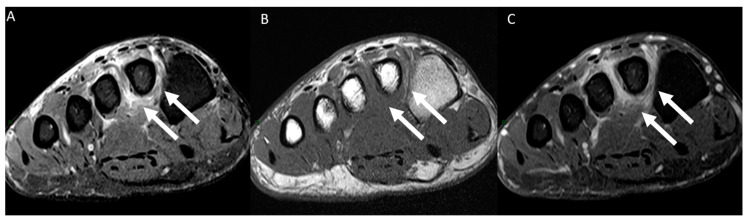
A 36-year-old man presented with right foot pain persisting for 2 days. Forefoot MRI images in the short-axis plane, including a T2-weighted fat-suppressed scan (**A**), a T1-weighted scan (**B**), and a post-gadolinium T1-weighted fat-suppressed scan (**C**), revealed thrombosis in the communicating veins (arrows). This thrombosis was characterized by venous dilation, intraluminal thrombus and an intravenous filling defect.

**Figure 7:**
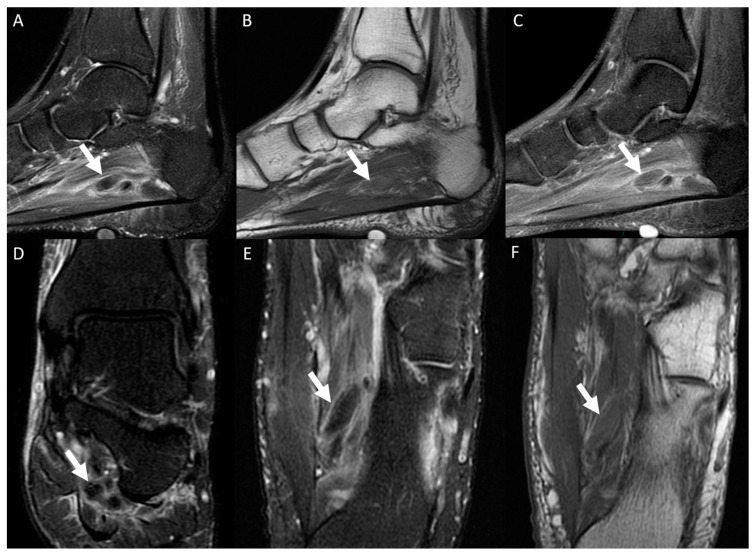
A 45-year-old woman presented with right ankle plantar pain persisting for 2 days, with a clinical suspicion of plantar fasciitis. Ankle MRI images, including a sagittal T2-weighted fat-suppressed scan (**A**), a T1-weighted scan (**B**), and a post-gadolinium T1-weighted fat-suppressed scan (**C**), as well as a coronal T2-weighted fat-suppressed scan (**D**), a short-axis plane T2-weighted fat-suppressed scan (**E**), and a T1-weighted scan (**F**), revealed extensive thrombosis of the lateral plantar vein (arrows). This thrombosis exhibited venous dilation, intraluminal thrombus, and an intravenous filling defect.

The following data were tabulated: the anatomical location of MRI examination (ankle or forefoot); laterality (right or left); the use of intravenous contrast medium (yes or no); the vein(s) affected (plantar digital veins, plantar metatarsal veins, plantar venous arch, communicating vein, lateral plantar vein, medial plantar vein, common/single plantar vein, posterior tibial vein, or other); and the imaging findings for the affected veins (perivascular edema, muscle edema, vascular ectasia/dilation, intraluminal signal characteristics in T1 and T2 fat-saturated [high, intermediate, or low], and, if intravenous contrast medium was used, perivascular enhancement and intravenous filling defect). Figure 1 shows the plantar venous anatomy and Figure 2, Figure 3, Figure 4, Figure 5, Figure 6 and Figure 7 illustrate different cases of PVT diagnosed by MRI. (Figure 1, Figure 2, Figure 3, Figure 4, Figure 5, Figure 6 and Figure 7).

### 2.6. Statistical Analysis

Summary measures (mean, standard deviation, median, minimum, and maximum) were used to describe the collected data. Absolute and relative frequencies, as well as associations, were assessed using the χ^2^ test or likelihood ratio test. Interobserver agreement was evaluated using the kappa coefficient. Data analyses were conducted using IBM-SPSS for Windows version 22.0 software, and Microsoft Excel 2010 software was utilized for data tabulation. The significance level for all the tests was set at 5%. All analyses were conducted by a senior statistician.

## 3. Results

### 3.1. Clinical Data

A total of 142 MRI exams were identified, with reports mentioning the diagnosis of thrombosis. However, 12 of these cases were excluded upon consensus among the readers because the findings were not consistent with PVT. The remaining 130 MRI scans corresponded to a total 112 patients since some underwent the concurrent MRI of the ankle and forefoot. Specifically, there were 86 forefoot exams and 44 ankle exams.

A total of 47 out of 112 (42%) of the patients were female, and 65 out of 112 (58%) were male; the majority of the exams (106/112—96.6%) were performed using a 1.5 T MRI scanner. Most of the studies (78/130, 60%) were of the left foot.

Thrombosis was detected solely in the forefeet of 68 patients (60.7%) and solely in the ankles of 26 (23.2%) patients. Additionally, 18 (16.1%) patients presented PVT in both the forefoot and ankle. With regard to the patients who underwent the MRI of the ankle and forefoot, in all cases, PVT was visualized in both the scans. Notably, no cases of bilateral PVT were observed in any patient, and there were no patients with bilateral exams. Further details regarding the demographic and clinical distribution of the patients can be found in Table 1. None of these clinical or demographic data were associated with the presence of PVT. With regard to sports practice, few patients answered the questionnaire about this information, so it was not possible to analyze the association with PVT.

### 3.2. Distribution of MRI Findings

Table 2 shows the anatomical distribution and frequency of veins affected by PVT observed in our cohort.

Table 3 shows the PVT features observed using MRI, being presented for the entire foot and for the ankle and forefoot separately. The *p*-values were calculated to assess the statistical differences between the findings from the forefoot vs. ankle groups. The findings of muscle edema and T2 signal alteration were significantly more prevalent in the ankle than they were in the forefoot. With regard to additional findings, seven cases (8.1%) of metatarsal stress fracture were observed in the forefoot examinations, and one case (2.3%) of fibula stress fracture in the ankle examinations, with no statistical association between the fractures and PVT. The remainder of the parameters evaluated also did not show differences regarding location.

### 3.3. Interobserver Agreement

Table 4 shows the kappa correlation coefficient between the readers for each parameter analyzed. The variables “perivascular edema”, “vascular ectasia” and “perivascular enhancement” were seen in 100% of the cases by both the evaluators, with the kappa value corresponding to 1.0 (perfect agreement). The signal intensity on the T1- and T2-weighted images (high, intermediate, or low) showed an agreement of 0.05. The remaining parameters (varied PVT locations and muscle edema) ranged from 0.16 to 0.71. PVT affecting the lateral plantar vein showed substantial agreement between the readers (0.71).

## 4. Discussion

Our study showed an overview of the most common anatomical locations and MRI features for PVT affecting the ankle and forefoot, with the main sites of involvement being the lateral plantar veins and plantar metatarsal veins. As for the reading parameters, venous ectasia, edema, and perivascular enhancement were found in all the cases, followed by an intravenous filling defect, which was frequently present.

PVT is a condition that has not been extensively explored in the literature, with a few studies published [2,3,6,17,18,19,20,21,22,23,24]. Legrand et al. [24] was the first to describe it in a case report in 1997. Miranda et al. [4] presented a retrospective case series of 20 patients, showing the MRI findings of PVT. Czihal et al. [5] presented a retrospective case series with 22 patients diagnosed using US and MRI. Barros et al. [19] reported a case series with 11 patients diagnosed with US. In 2021, Edwards et al. [2], in a review, showed that 44 cases of PVT had been reported in the literature up to that point, in 10 papers. They were diagnosed either by US or a combination of US and MRI; however, most of these cases involved the ankle. Therefore, to our knowledge, our study comprising 112 patients with a total of 130 positive MRI studies for PVT presents the largest sample to date.

Patients commonly report uncharacteristic moderate-to-severe foot pain [1,2,5], making clinical diagnosis challenging, and leading to the consideration of various other differentials, including, but not limited to, intermetatarsal bursitis, Morton’s neuroma, sesamoiditis, plantar fasciitis, tendon pathologies, ganglion cysts, and stress fractures. Consequently, physicians rarely suspect PVT as the cause of pain [1,25]. The diagnosis in all our cases was initially suggested using MRI, with only three patients undergoing the examination due to the clinical suspicion of PVT. This lack of suspicion likely stems from the difficulty in diagnosing and the limited awareness of this condition, even among orthopedists, podiatrists, and other medical specialists.

Regarding the clinical and demographic data from our study, we did not find that the factors such as gender, age and BMI act as risk factors for this condition, which is in line with the literature reviewed. According to Rastel et al., the duration between the onset of symptoms and diagnosis can vary, sometimes taking up to four weeks, but averaging around one week in the majority of cases [10]. In our series, this interval ranged from 1 day to 240 days, with a mean of 29.1 days. This variability could be attributed to many factors, including misdiagnosis or late diagnosis attributed to the non-specific nature of plantar pain, potentially leading to delayed medical attention, and possibly contributing to the development of more extensive PVT in certain cases.

The pathogenesis of PVT remains uncertain. The cause could be idiopathic or related to systemic or local factors [1,6,7,8]. The “Virchow triad” (venous ectasia, endothelial damage, and inflammation) is the traditional explanation for the pathophysiological mechanism behind the development of DVT [8,9]. The possible predisposing factors include coagulation disorders, oral contraceptives, infection, malignancy, airplane travel, trauma, mechanical stress, athletic activity, recent surgery, pos-operative immobilization, and pressure from orthotics [1,2,6,7]. However, up to 50% of cases can be idiopathic [5].

The anatomy of the plantar veins is highly variable and complex, with many anatomical variations described. In a simplified distal-to-proximal description, the plantar digital veins originate from the venular plexus on the toes, and subsequently merge to form the metatarsal veins. These veins then contribute to the formation of the deep venous arch. From this point, the medial and lateral plantar veins arise, converging behind the medial malleolus to form the posterior tibial veins [1,4,6,26,27].

When considering all the foot veins, our analysis revealed that the lateral plantar veins were the most involved, present in 53.1% of our cases. Specifically, we identified thrombosis in the medial plantar vein in 3.8% of all the cases and in 9.1% of the cases when considering only the ankle veins. These observations are similar to the literature, which also suggests a higher frequency of the involvement of the lateral plantar veins when compared to the medial veins [1,4,5]. This may be attributed to increased mechanical load or stress on the plantar region of the foot, which appears to be a very typical presentation of PVT [17]. The proximity of the lateral plantar veins to the sole of the foot may render them more susceptible to such stressors compared to medial plantar veins [1]. Furthermore, the lateral plantar veins follow a more muscular course between the foot muscles, while the medial plantar veins have a more tendinous course [28]. This anatomical difference can also make the lateral plantar veins more prone to mechanical strain, compression, and venous stasis. Additionally, medial plantar veins are also smaller, making thrombosis potentially more challenging to detect [1].

In our study, when considering only the veins of the forefoot, the plantar metatarsal veins were the most affected (68.6%), followed by the plantar venous arch (46.5%) and by the plantar communicating veins (37.2%). As also noticed by Edwards et al. [2], we observed the concurrent involvement of the plantar metatarsal veins and the plantar venous arch. Specifically in the forefoot, the plantar lateral veins were affected in 30.2% of the cases, and the medial plantar veins were affected in only 1.2% of the cases. When focusing exclusively on the veins of the ankle, the lateral plantar veins were affected in almost all the cases (97.7%), with many cases presenting extension into the common plantar veins (70.5%) and the posterior tibial veins (43.2%).

Perforating veins play a crucial role in the venous drainage of the foot, serving as an ascending venous pump, making the connection between the deep veins and the superficial dorsal venous arch [28,29,30]. Among these, the first interspace metatarsal perforator is the primary connector between the deep veins and the superficial veins and could potentially serve as the origin of PVT [1,28]. According to Rastel et al., the first interspace metatarsal perforator is the most frequently affected perforator vein in cases of PVT [10]. In our cases, we observed a notable number of PVT cases topographically related to the second metatarsal, involving especially the first interspace metatarsal and the metatarsal vein located between the first and second metatarsals, although we did not quantify these veins individually. This aspect could be further investigated in a future study.

Some of the patients engaged in sports activities such as running, which contributes to the possibility of repetitive trauma in the plantar region as a causal factor [4]. According to Czihal et al., in a series involving 22 patients, a history of mechanical strain to the foot was reported in 32% of the cases [5]. In our series, seven patients had an associated metatarsal stress fracture, but whether this is a causal association cannot be determined. The same author reported that recurrence of PVT is not uncommon, occurring in 27% in their series, and it can manifest again as PVT [5]. In our series, only one patient had the recurrence of PVT one year later.

MRI has proven to be particularly beneficial in assessing the thrombosis of the foot [2], primarily due to its superior detail and resolution [1]. MRI can be highly valuable in diagnosing PVT, particularly when clinical suspicion includes any of the differentials for pain in the foot plantar region. The growing utilization of MRI for assessing either acute or chronic foot pain resulted in PVT being initially diagnosed first with MRI in our setting. The main advantage of MRI is its capability to reliably diagnose this condition and rule out the other causes of plantar pain.

The MRI findings for PVT commonly include perivascular edema, muscular edema, intravascular heterogeneous signal intensity, venous ectasia, the presence of collateral veins, perivascular enhancement, and intravenous filling defects [1,4]. Notably, perivenous edema and enhancement emerge as the most prominent findings, correlating with inflammatory soft tissue changes [25]. In our study, muscle edema was observed in 81.4% of the forefoot exams and in 95.5% of ankle exams, which may be attributed to the larger muscle groups in the ankle region. Perivascular edema was present in 100% of the cases. In relation to the analysis of muscle edema and perivascular edema, these findings can overlap, with some cases in which only perivascular edema was valued, but not muscle edema. This may explain the lower level of agreement between the evaluators in this study regarding muscle edema and perfect agreement regarding perivascular edema. Along with increasing the awareness of this entity, radiologists should actively assess plantar veins in every foot MRI examination, especially in the forefoot, where we believe MRI is superior to US in the evaluation of PVT. Additionally, evaluating forefoot PVT using US can be challenging due to the proximity of osseous structures and the thickness of the plantar skin and subcutaneous layer. These factors result in artifacts and the attenuation of the US beam [1,6,30].

The signal intensity within the vessel lumen can vary and may appear increased or decreased on T1- or T2-weighted images due to the diverse imaging characteristics of blood and its products. Additionally, intravascular signal intensity is influenced by the blood flow motion [31]. The signal characteristics on T1- and T2-weighted images were highly variable, a phenomenon that can be attributed to the diverse imaging characteristics of blood and its products. Furthermore, another aspect that can limit the accuracy of signal intensity evaluation is that sometimes PVT may be extensive, and different evaluators may assess the signal in different regions of the vein. Faster blood flow at the center of the vein may mimic thrombus, resulting in a hypointense signal in the center of the vein. Typically, in exams without contrast, intravascular thrombus is best visualized on T2-weighted images, where it appears hypointense, replacing the normal hyperintense signal [1,31]. Intravenous contrast proves to be beneficial in detecting PVT, particularly in the forefoot, where the veins are smaller. Following contrast administration, thrombus can be delimitated as a filling defect, thereby enhancing the diagnostic accuracy [1,32]. Intravenous filling defects were seen in 97.7% of our sample, without differences observed between the ankle and forefoot veins. In uncertain cases, intravenous contrast may prove beneficial in diagnosis, particularly in the forefoot. Another aspect that demonstrates the benefit of intravenous contrast is in the differentiation between PVT of the metatarsal veins in the distal interdigital space and Morton’s neuroma. We encountered difficulty in distinguishing between these two conditions without postcontrast enhanced sequences.

Although venous ectasia is not regarded as a reliable diagnostic criterion for PVT, as it can also be observed in patients without PVT, such a finding was present in 100% of our cases. Besides PVT, venous dilation may be due to multiple reasons, such as anatomic variations, or be secondary to venous insufficiency and varicosities [1].

The main strength of our study is that it brings the largest series of PVT to date, particularly the significant number of forefoot PVT cases, allowing us to present a comprehensive assessment of the PVT features on MRI. However, the limitations include its retrospective nature, the lack of correlation with ultrasound (US), and the absence of patient follow-ups. In addition, we did not have detailed access to the patients’ sporting activities to verify the possible causative associations. Therefore, future studies with a prospective design and longitudinal data are encouraged to further advance understanding PVT.

## 5. Conclusions

The present study evaluated the findings of PVT diagnosed by MRI, describing the frequency of the most affected veins and its imaging characteristics. When considering the entire foot or just the ankle, the lateral plantar veins were most frequently affected (53.1% and 97.7%, respectively). For the forefoot alone, the plantar metatarsal veins were most frequently affected (68.6%). As for the imaging findings, venous ectasia, edema, and perivascular enhancement were present in 100% of the cases, followed by an intravenous filling defect, which was observed in 97.7% of the cases. In the assessment of painful conditions of the plantar region of the foot, it is imperative to consider PVT as a potential diagnosis. PVT appears to be a less common variant of a more proximal deep venous thrombosis, which is a more prevalent condition, sharing similar risk factors. However, mechanical load and stress appear to be a unique risk factor specifically associated with PVT. As we aim to increase the awareness of this pathology and corresponding MRI features, this condition may become more readily recognized.

## Figures and Tables

**Table 1:** Demographic and clinical data of the 112 analyzed patients.

Variable	Mean	SD	Median	Minimum	Maximum
Age (years)	50.2	14.5	48.5	18	82
BMI (kg/m^2^)	25.6	3.6	25.3	19.1	38.0
Time of symptoms (days)	29.1	43.3	14	1	240

SD: standard deviation. BMI: body mass index.

**Table 2:** Anatomical distribution and frequency of PVT.

Anatomical Location	Forefoot	Ankle	Total (Forefoot and Ankle)
Plantar digital veins	22/86 (25.6%)	0/44 (0%)	22/130 (16.9%)
Plantar metatarsal veins	59/86 (68.6%)	0/44 (0%)	59/130 (45.4%)
Plantar venous arch	40/86 (46.5%)	10/44 (22.7%)	50/130 (38.5%)
Communicating/perforating veins	32/86 (37.2%)	1(44) (2.3%)	33/130 (25.4%)
Lateral plantar vein	26/86 (30.2%)	43/44 (97.7%)	69/130 (53.1%)
Medial plantar vein	1/86 (1.2%)	4/44 (9.1%)	5/130 (3.8%)
Common plantar vein	0/86 (0%)	31/44 (70.5%)	31/130 (23.8%)
Posterior tibial vein	0/86 (0%)	19/44 (43.2%)	19/130 (14.6%)

PVT: plantar vein thrombosis.

**Table 3:** MR imaging findings of PVT.

Variable	Total (Forefoot and Ankle)	Forefoot	Ankle	*p* Value (Forefoot vs. Ankle)
Muscular edema	112/130 (86.2%)	70/86 (81.4%)	42/44 (95.5%)	*p* = 0.028
Intravascular signal characteristics on T1-weighted images	High 24/130 (18.5%)	High 13/86 (15.1%)	High 11/44 (25.0%)	*p* = 0.087
	Intermediate 102/130 (78.5%)	Intermediate 69/86 (80.2%)	Intermediate 33/44 (75%)	
	Low 4/130 (3.0%)	Low 4/86 (4.7%)	Low 0/44 (0%)	
Intravascular signal characteristics on T2-weighted images	High 108/130 (83.1%)	High 68/86 (79.1%)	High 40/44 (90.9%)	*p* = 0.044
	Intermediate 14/130 (10.8%)	Intermediate 13/86 (15.1%)	Intermediate 1/44 (2.3%)	
	Low 8/130 (6.1%)	Low 5/86 (5.8%)	Low 3/44 (6.8%)	
Perivascular edema	130/130 (100%)	86/86 (100%)	44/44 (100%)	*p* > 0.999
Perivascular enhancement	130/130 (100%)	86/86 (100%)	44/44 (100%)	*p* > 0.999
Intravenous filling defect	127/130 (97.7%)	84/86 (97.7%)	43/44 (97.7%)	*p* > 0.999
Venous ectasia	130/130 (100%)	86/86 (100%)	44/44 (100%)	*p* > 0.999

PVT: plantar vein thrombosis.

**Table 4:** Kappa index for each variable studied in all exams.

Variable	Kappa (CI 95%)	Sensitivity (CI 95%)	Specificity (CI 95%)	PPV (CI 95%)	NPV (CI 95%)
PVT in the plantar digital veins	0.56 (0.348; 0.764)	45.5 (24.4; 67.8)	99.1 (94.9; 100)	90.9 (58.7; 99.8)	89.9 (83; 94.7)
PVT in the plantar metatarsal veins	0.62 (0.489; 0.751)	91.5 (81.3; 97.2)	71.8 (59.9; 81.9)	73 (61.4; 82.6)	91.1 (80.4; 97)
PVT in the plantar venous arch	0.408 (0.255; 0.561)	42 (28.2; 56.8)	95 (87.7; 98.6)	84 (63.9; 95.5)	72.4 (62.8; 80.7)
PVT in the communicating vein	0.23 (0.044; 0.416)	30.3 (15.6; 48.7)	89.7 (81.9; 94.9)	50 (27.2; 72.8)	79.1 (70.3; 86.3)
PVT in the lateral plantar vein	0.71 (0.592; 0.828)	76.8 (65.1; 86.1)	95.1 (86.3; 99)	94.6 (85.1; 98.9)	78.4 (67.3; 87.1)
PVT in the medial plantar vein	0.585 (0.217; 0.953)	75 (19.4; 99.4)	97.6 (93.2; 99.5)	50 (11.8; 88.2)	99.2 (95.6; 100)
PVT in common plantar vein	0.227 (0.058; 0.396)	16.1 (5.5; 33.7)	100 (96.3; 100)	100 (47.8; 100)	79.2 (71; 85.9)
PVT in the posterior tibial vein	0.355 (0.114; 0.596)	26.3 (9.2; 51.2)	99.1 (95.1; 100)	83.3 (35.9; 99.6)	88.7 (81.8; 93.7)
Muscular edema	0.162 (−0.04; 0.364)	83.8 (75.6; 90.1)	35.3 (14.2; 61.7)	89.4 (81.9; 94.6)	25 (9.8; 46.7)

PVT: plantar vein thrombosis. CI = confidence interval. PPV = positive predictive value. NPV = negative predictive value.

## Data Availability

The original contributions presented in the study are included in the article. Further inquiries can be directed to the corresponding author.
